# Comparative Study of Physicochemical and Antibacterial Properties of ZnO Nanoparticles Prepared by Laser Ablation of Zn Target in Water and Air

**DOI:** 10.3390/ma12010186

**Published:** 2019-01-08

**Authors:** Ekaterina A. Gavrilenko, Daria A. Goncharova, Ivan N. Lapin, Anna L. Nemoykina, Valery A. Svetlichnyi, Ali A. Aljulaih, Neli Mintcheva, Sergei A. Kulinich

**Affiliations:** 1 Siberian Physical Technical Institute, Tomsk State University, Lenina, 36, Tomsk 634050, Russia; gavrilenko2470@gmail.com (E.A.G.); dg_va@list.ru (D.A.G.); 201kiop@mail.ru (I.N.L.); 2 Laboratory of Biopolymers and Biotechnology, Tomsk State University, Lenina, 36, Tomsk 634050, Russia; nemoykina@rambler.ru; 3 Institute of Innovative Science and Technology, Tokai University, Hiratsuka, Kanagawa 259-1259, Japan; ali.aljulaih@gmail.com (A.A.A.); nnmintcheva@mgu.bg (N.M.); 4 Department of Mechanical Engineering, Tokai University, Hiratsuka, Kanagawa 259-1259, Japan; 5 Department of Chemistry, University of Mining and Geology, 1700 Sofia, Bulgaria; 6 Research Institute of Science and Technology, Tokai University, Hiratsuka, Kanagawa 259-1259, Japan

**Keywords:** pulsed laser ablation in water, pulsed laser ablation in air, ZnO nanoparticles, biomedical materials, PLLA-scaffold, antibacterial properties

## Abstract

Here, we report on ZnO nanoparticles (NPs) generated by nanosecond pulsed laser (Nd:YAG, 1064 nm) through ablation of metallic Zn target in water and air and their comparative analysis as potential nanomaterials for biomedical applications. The prepared nanomaterials were carefully characterized in terms of their structure, composition, morphology and defects. It was found that in addition to the main wurtzite ZnO phase, which is conventionally prepared and reported by others, the sample laser generated in air also contained some amount of monoclinic zinc hydroxynitrate. Both nanomaterials were then used to modify model wound dressings based on biodegradable poly l-lactic acid. The as-prepared model dressings were tested as biomedical materials with bactericidal properties towards *S. aureus* and *E. coli* strains. The advantages of the NPs prepared in air over their counterparts generated in water found in this work are discussed.

## 1. Introduction

Because of their unique physicochemical properties, nanomaterials have recently attracted a lot of research interest as materials and components for various applications. Zinc oxide (ZnO) is a wide-bandgap semiconductor of the II-VI group (E_g_ = 3.3 eV) [1,2]. Of all known metal-oxide semiconductors, it is probably the most extensively studied material with applications in numerous fields, such as optoelectronics, piezoelectronics, spintronics, solar energy, gas sensing, bio-sensing, UV-blue diodes, and photocatalysis, just to name several [1,2,3,4,5,6]. Because of its high surface energy, its nanoparticles (NPs) are able to generate various reactive oxygen species (ROS), which makes such NPs efficient bacteria inhibitors that are attractive for biomedical use, as well. Having low toxicity, ZnO nanomaterials, unlike those of many other semiconductor oxides, can be applied to problems such as water, working surface and wound disinfection, as well as in food industry, and hence the number of studies on potential use of ZnO nanomaterials keeps growing quite fast nowadays [1,2,3,4,5,6]. 

There are many approaches to preparing ZnO NPs, including, for example, hydrothermal methods, chemical deposition from gas phase, microwave and sonochemical approaches, numerous sol-gel-based methods, and so on [1,2,3]. One attractive method to produce so-called “pure” ZnO nanostructures (i.e., with surface free of any impurities or stabilizers) for biomedical use is based on pulsed laser ablation (PLA) of metallic Zn target in different media (mainly in liquids) [4,5,6,7,8,9,10]. The method uses pulsed lasers (with different pulse energy and pulse width) to ablate zinc target and produce species which then react with the surrounding liquid and form ZnO NPs. The formation mechanisms are quite complex, depending on laser pulse parameters and composition of liquid medium, and involve several fast stages that overlap and compete with each other, such as: absorption of irradiation, melting and evaporation on the target, expansion of the formed plasma/vapor, chemical reactions and quenching by the liquid, formation of clusters and primary NPs, and secondary irradiation of the formed NPs by laser beam, just to name the main ones. Thus, when applied in liquid phase, the method combines both “top-down” and “bottom-up” synthesis approaches, as the target gets broken down by the laser beam into very small species (particles, clusters, atoms, radicals, ions), after which such species get oxidized and quenched to ambient temperature as ZnO NPs [7,9]. In addition to PLA in liquids [4,5,6,7,8,9,10], more recently, the PLA approach in gases or vacuum has also been applied in nanomaterial preparation, including Zn-based NPs [11,12]. In the case of PLA in air [13]: (1) target surface temperature increases, reaching melting and vaporization points; (2) plasma is formed due to interaction between the laser pulse and vaporized material; (3) ZnO NPs are formed in the plasma plume as a result of reactions between excited zinc species and ionized oxygen molecules from air, as well as outside the plume due to diffusion, where plasma ions interact with unexcited oxygen molecules. Finally, the formed ZnO NPs are deposited onto the substrate.

The characteristics of laser-produced ZnO NPs were found to depend on both laser parameters (wavelength, frequency, pulse energy, energy fluence, and pulse duration) and on target and ablation medium (vacuum, gas, or liquid). The effect of liquid on the physicochemical properties of generated ZnO NPs has been extensively studied, with the main media used being based on water, water-ethanol mixtures, H_2_O_2_, alkali and acidic solutions, salts and various surfactants [4,5,6,7,8,9,10,14,15,16]. The produced NPs were reported to have different shapes, such as rods, spheres, flakes, dendrites, spindles, also including additional phases, such as β-Zn(OH)_2_, metallic zinc, zinc peroxide ZnO_2_, or having core@shell morphology Zn@ZnO as a result of incomplete oxidation of metallic zinc [8,14,15,16].

The PLA-generated NPs were reported to demonstrate high reactivity, and unique optical, catalytic and antibacterial properties, which is often explained by their structural and surface defects [4,5,6,7,8,10,14,15,16]. The defectiveness of various ZnO NPs has been extensively analyzed, as various defects are known to provide different functionalities to ZnO materials [6,14,17,18]. Similar to other II-VI semiconductors, ZnO tends to have deficiencies in its anion sub-lattice, which leads to the formation of oxygen vacancies (V_O_) with low formation energy, interstitial zinc (Zn_i_) and zinc atoms in oxygen sub-lattice (Zn_O_), as well as defects such as interstitial oxygen (O_i_) and oxygen in the zinc sub-lattice (O_Zn_), the latter defects requiring high energies [15,17]. In their comparative study, Goto and co-workers showed that PLA in pure water mainly generated ZnO NPs with O_i_ defects, while NPs prepared in pure ethanol were rich in Zn_i_–V_Zn_ defects, which was explained by the stronger oxidation ability of water [15]. At the same time, no information on defect composition of ZnO NPs PLA prepared in air has been reported thus far.

The present work aimed at preparing ZnO NPs by means of PLA in water and air and comparing their composition, structure, and properties. The materials were then incorporated into polymeric tissues based on poly l-lactic acid (PLLA) used as scaffold, where their antibacterial behavior against two different bacteria strains was evaluated and compared. 

## 2. Materials and Methods

### 2.1. Preparation of ZnO NPs Using PLA Method

**Figure 1:**
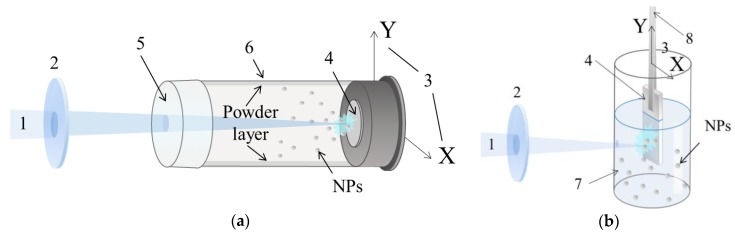
Setups used in the present study for PLA in air (**a**) and water (**b**). 1: laser beam; 2: focusing lens; 3: movement direction for target; 4: Zn target; 5: polyethylene membrane; 6: cylindric reactor: 7: distilled water; 8: target holder.

In this study, all nanomaterials were obtained by means of a nanosecond pulsed laser (Nd:YAG type, LOTIS TII, model LS-2131M-20, Minsk, Belarus) that ablated metallic Zn targets (purity 99.5%) using the following parameters: 1064 nm, 20 Hz, 7 ns, and 150 mJ/pulse (as wavelength, frequency, pulse width and pulse energy, respectively). Two somewhat different setups were used, as presented in Figure 1.

For experiments in air, target with a diameter of 30 mm and thickness of 10 mm was fixed onto the back wall of a cylindrical quartz reactor filled with ambient air (see Figure 1a). The reactor was 200 mm in length, its internal diameter being 45 mm, and its volume being ~300 cm^3^. The wall of the reactor was made of polyethylene membrane which is transparent for the irradiation used. The laser beam was focused on the target surface by a long-focus (F = 500 mm) collecting lens. The produced NPs precipitated on the reactor walls forming a powdery layer. The whole ablation procedure took 3 h, during which the target was slowly moved along the vertical direction. The powder was then mechanically removed from the walls, and the collected sample was denoted as ZnO_air.

To prepare ZnO nanostructures in liquid phase, a zinc plate target (40 × 15 × 10 mm^3^ in size) was immersed into a cylindrical glass reactor filled with distilled water. The laser irradiation was focused by a short-focus (F = 50 mm) lens, with the beam entering the reactor through its sidewall, as seen in Figure 1b. To enhance NP oxidation, air was bubbled through the water by a compressor. The ablation time was 2 h, after which the produced colloid was centrifuged and dried in air at 50 °C. The ablation and separation procedures were repeated several times to collect a sufficient amount of nanomaterial. Hereafter, the sample produced in water is denoted as ZnO_water.

In both cases, the initial power density on the target surface was estimated to be around 250 MW/cm^2^. To maintain uniform irradiation of the target surface, thus providing uniform and constant ablation, the target was automatically moved both horizontally and vertically in the XY plane normal to incident beam. In both cases, the average material production rate observed after experiments was ~40 mg/h. More detailed description of similar experiments both in liquid and air have previously been published elsewhere [18].

### 2.2. Preparation of ZnO-PLLA Composites

As a model material for bandage tissues with antibacterial properties, we chose biodegradable polymer poly l-lactic acid (PLLA), which easily decays upon hydrolysis and fermenting processes giving rise to non-toxic compounds. PLLA is also known to be biocompatible, which makes it an excellent candidate for pharmaceutical and biomedical applications [19]. Using the previously reported methodology based on electrospinning [20], PLLA scaffold was prepared and then kindly supplied to us by Drs. S. Tverdokhlebov and E. Bolbasov (Tomsk Polytechnic University). 

The abovementioned nanopowders of ZnO (samples ZnO_air and ZnO_water) were dispersed in distilled water by means of sonication, so that the concentration of materials was 1 g/L in both cases. Pieces of PLLA scaffold, 10 × 10 cm^2^ in size and 250 μm thick, were placed on a net where they were homogenously fed with colloidal solutions with ZnO NPs by means of a pump. The NP-loaded scaffolds were then dried with air at 20 °C, after which the procedures were repeated until the loading level of 1 mg/cm^2^ was achieved. Thus, two samples were obtained that consisted of PLLA scaffolds loaded with ZnO NPs of two types with the same loading (composite samples referred to as ZnO_water_PLLA and ZnO_air_PLLA below). To confirm whether the loaded NPs were strongly attached to the PLLA matrix, the prepared composites were washed with distilled water and the obtained water was further analyzed using UV-vis spectroscopy. No intense absorption of ZnO NPs was detected, implying good adhesion of ZnO NPs on the PLLA fibers.

### 2.3. Characterization of Nanopowders and ZnO-PLLA Composites

The crystal structure of the samples was analyzed by X-ray diffractometry (XRD), for which a XRD 6000 model (from Shimadzu, Kyoto, Japan) was used. The samples were analyzed with CuKα irradiation (λ = 1.54056 Å) at a sampling rate of 0.02°/s and in the range of 2θ from 10 to 70°. Phase identification and quantitative analysis of XRD patterns were conducted using the database PDF4 and PowderCell 2.4 software (from BAM, Berlin, Germany). The morphology and size of NPs were studied by transmission electron microscopy (TEM, model CM 12 from Philips, Eindhoven, The Netherlands) with accelerating voltage of 120 kV. Drops of freshly prepared dispersions were placed onto copper grids coated with carbon film and then dried at room temperature. The surface morphology of PLLA scaffolds loaded with ZnO NPs was studied by scanning electron microscopy (SEM, model VEGA 3 SBH from Tescan, Brno, Czech Republic).

The specific surface area of the prepared powder samples was evaluated by using a standard Brunauer–Emmett–Teller procedure (low-temperature adsorption/desorption of nitrogen) in a TriStar II 3020 analyzer (Micromeritics, Norcross, GA, USA). The samples were degassed at 200 °C for 2 h prior to the measurements in a VacPrep 061 station (Micromeritics, Norcross, GA, USA).

UV-Vis absorption spectra of both powder samples and ZnO-PLLA composites were examined by the diffuse-reflection spectroscopy (DRS) technique on a Cary 100 spectrophotometer (Varian, Australia) with accessory DRA-CA-30I (Labsphere, North Sutton, NH, USA) in the range of 200–800 nm. As reference samples, MgO powder and as-supplied (i.e., unloaded with ZnO) PLLA scaffold were used. Reflection spectra were converted using the Kubelka–Munk transformation approach. The obtained absorption spectra were used to evaluated the bandgap values (E_g_) of the ZnO nanomaterials, for which they were replotted in the (F(R)hυ)*^n^* versus E(eV) coordinates and the absorption edge was then extrapolated onto the absciss axis (where y = 0) in accordance with the Tauc method [21]. Here *n* depends on the nature of electronic transition and is 1/2 for indirect and 2 for direct semiconductors with crystalline structure. For ZnO, *n* = 2 as it is a direct semiconductor. Photoluminescence (PL) spectra of the samples were recorded at room temperature by means of a Fluorolog 3-22 spectrometer (Horiba, Jobin Yvon, Edison, NJ, USA) with an excitation wavelength of 350 nm. Fourier-transform infrared (FTIR) spectra were registered by a Nicolet 6700 spectrometer (Thermo Fisher Scientific, Waltham, MA, USA). Raman shift spectra were collected by an InVia (Renishaw, Gloucestershire, UK) Raman spectrometer, while the second harmonic of Nd:YAG laser (λ = 532 nm) was used as the excitation source. 

Zeta potential values of the NPs were determined through electrophoretic light scattering with phase analysis (PALS) using a Nano Brook Omni instrument (Brookhaven, NY, USA). Titration was done with aqueous KOH, prior to which ZnO dispersions in water (0.1 g/L) were sonicated for 10 min.

### 2.4. Antibacterial Activity of NPs

The antibacterial activity of the prepared inorganic-organic composites as model wound dressing materials was tested in accordance with the standard ISO 20743:2013 [22] and by using two bacteria strains: (1) gram-positive *Staphylococcus aureus* (*S. aureus*, test strain ATCC 25923) and (2) gram-negative *Escherichia coli* (*E. coli*, test strain B-6954, Russian Collection of Microorganisms). Both loaded with NPs and as-supplied (ZnO-free) PLLA scaffolds were cut to samples with sizes 3 × 5 cm^2^ for testing. Bacteria cultures (0.2 mL) with concentration of 10^5^ CFU/mL in nutrient broth (diluted 20 times with distilled water) were placed onto each PLLA sample. Immediately after contact, as well as after 24 h, the tested objects were rinsed with 20 mL of physiological solution for 5 min. The obtained liquid was placed into a Petri dish containing 100 mL of beef-extract agar (also known as meat-and-peptone agar, MPA), and the obtained material was then cultivated at 37 °C for 24 h. The quantity of grown microorganisms was counted in cultures extracted after the contact with both control (ZnO-free) and NP-modified PLLA samples after cultivation, which permitted to determine the death percentage of tested microorganisms. The values of antibacterial activity A were determined using Formula (1):

A = (lgC_t_ − lgC_0_) − (lgT_t_ − IgT_0_) = F − G, (1)

where F = (lgC_t_ − lgC_0_) is the growth rate on the control (ZnO-free) PLLA sample; lgC_t_ is the average decimal logarithm of the number of bacteria found on three control samples incubated for 24 h; lgC_0_ the average decimal logarithm of the number of bacteria observed on three control samples immediately upon seeding with bacteria; G = (lgT_t_ − lgT_0_) the growth rate on the sample loaded with antibacterial NPs; lgT_t_ the average value of decimal logarithm of the number of bacteria observed after incubation for 24 h on three treated samples; and lgT_0_ the average decimal logarithm of bacteria number observed immediately after bacteria seeding on three PLLA samples loaded with ZnO.

## 3. Results and Discussion

### 3.1. XRD Data

**Figure 2:**
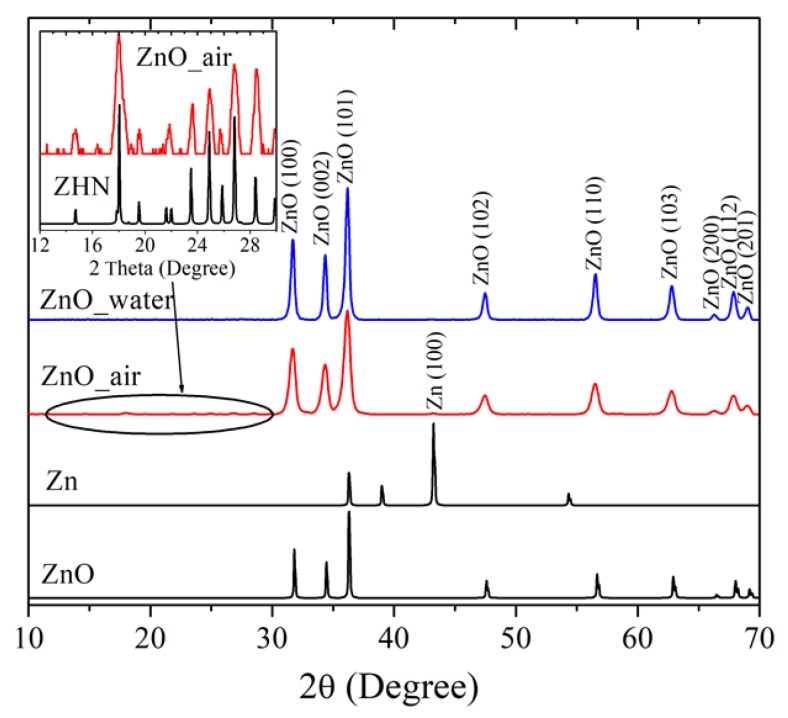
XRD patterns of PLA-prepared samples ZnO_water (blue), ZnO_air (red), and Zn and ZnO patterns from database (black). Inset shows pattern of sample ZnO_air and that of ZHN phase in a narrower range of 2θ between 12° and 30°.

Figure 2 exhibits XRD patterns of the laser-produced samples ZnO_water (blue line) and ZnO_air (red) and compares them with XRD data for metallic Zn and ZnO, both taken from databases (black lines). Both samples are clearly seen to demonstrate intense peaks at 2θ = 31.68°, 34.34°, 36.17°, 47.48°, 56.51°, 62.78°, 66.27°, 67.85° and 69.00°, implying the presence of wurtzite ZnO (PDF4 Card no. 04-007-9805) as dominating phase. This is consistent with previous reports on ZnO NPs prepared via PLA in liquids [5,6,10,15,16], confirming that highly active Zn species generated by laser pulse react with oxygen and/or water molecules, giving rise to ZnO nanomaterial. The FWHM values of XRD peaks in pattern of sample ZnO_air were found to be larger than those of sampe ZnO_water, which is explained by smaller NP sizes of the former. Small amounts of metallic zinc were also detected in the samples, as very weak peaks at 2θ = 43.19° (PDF4 Card no. 01-071-4620), the signal being somewhat stronger in the sample produced in air than in its counterpart generated in water. This observation is also in agreement with previous studies as small amounts of metallic phase inclusion was previously reported by different groups for various ZnO NPs produced in liquids [6,14,16].

In addition to the diffraction peaks of ZnO, the sample produced in air exhibited a series of small peaks in the range 2θ = 10–30° (see inset in Figure 2). The latter peaks were indexed as monoclinic zinc hydroxynitrate (ZHN), Zn_5_(OH)_8_(NO_3_)_2_ × 2H_2_O, whose pattern (PDF4 Card no. 01-072-0627) is also given in the inset for convenience. Previously, this phase was reported in NPs generated via laser ablation of Zn foil immersed into aqueous solution of zinc nitrate [23]. In the present study, the formation of ZHN is explained by interaction of laser-induced plasma with Zn species (atoms, ions, radicals, clusters) and air components, such as molecules (N_2_, O_2_, H_2_O) and excited species (N^*^, NO^*^, NO_2_^*^, OH^*^). It is very likely that zinc nitrate Zn(NO_3_)_2_ is one of intermediate products of such reactions. It should be noted here that no formation of ZHN is observed as a result of atmospheric corrosion of metallic Zn, which supports the efficiency of PLA in preparing metastable phases. More detailed phase compositions of the ZnO samples, as well as their specific surface area and NP sizes, are presented in Table 1.

Thus, the XRD measurements indicate that the nanomaterial obtained in water was mainly ZnO with trases of metallic Zn, while the NPs prepared in air had a few per cent of ZHN.

### 3.2. Microscopic Observations

#### 3.2.1. TEM Images

**Figure 3:**
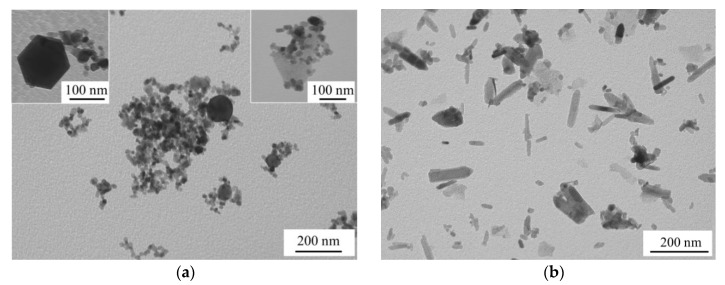
TEM images of samples: ZnO_air (**a**) and ZnO_water (**b**).

According to the TEM image of sample ZnO_air in Figure 3a, the material prepared in air consisted of NPs with different shapes: spheres, nanocubes, and polyhedrons (see insets in Figure 3a), with average sizes being 18–26 nm. Meanwhile, sample ZnO_water had mainly nanorods as its main component (Figure 3b). Since ZnO is well-known to be a high-surface-energy material if no surfactant is available, its NPs prepared in water at elevated temperatures were previously reported to agglomerate and recrystallize into nanorods [16,24]. Similarly shaped ZnO NPs were previously reported by Honda et al. [16] who ablated Zn in water with millisecond pulsed laser and explained nanorod formation by an increase in temperature during PLA. Another group also produced ZnO nanorods by PLA of Zn in water by means of nanosecond pulsed laser followed by heat treatment of the formed colloid at 60–80 °C [24]. In this study, we also used no surfactant when ablating in water, and thus some increase in temperature during PLA (and secondary irradiation of the initial ZnO NPs) could lead to their recrystallization into nanorods. ZnO NPs are known to have various shapes as the phase has no symmetry center in its crystal lattice [25]. This may explain the variety of shapes observed in Figure 3a as the NPs produced in air had no chance to agglomerate and recrystallize as nanorods. 

The sizes and shapes of NPs exhibited in Figure 3 correlate well with the results of BET measurements. The specific surface area of the smaller NPs produced in air is seen in Table 1 to be 1.8 times larger compared with their counterparts obtained in water. It is also worth noting one more time that the rod-shaped morphology and somewhat bigger sizes of the NPs presented in Figure 3b are results of secondary growth processes taking place in water after their initial formation.

#### 3.2.2. SEM Images of ZnO-PLLA Scaffolds

**Figure 4:**
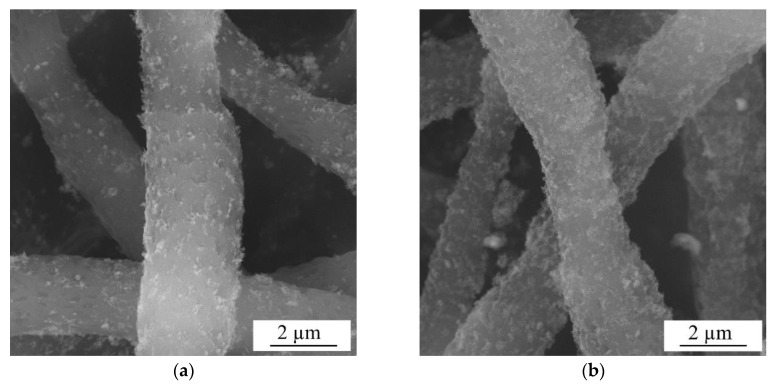
SEM images of model wound dressing tissues based on PLLA scaffold loaded with ZnO_water (**a**) and ZnO_air (**b**) NPs.

The surface morphology of PLLA scaffolds, both as-supplied and modified by ZnO NPs, was studied by SEM, with images of samples ZnO_water_PLLA (a) and ZnO_air_PLLA (b) being presented in Figure 4. It can be clearly seen in Figure 4 that both types of ZnO NPs are distributed quite uniformly over the scaffold surface. At the same time, the NPs obtained in water are seen in panel (a) to be somewhat more agglomerated, while those prepared in air cover the PLLA matrix somewhat more homogeneously. This can be explained by better dispersibility of sample ZnO_air in solvent prior to its loading onto the PLLA scaffold.

The uniform NP distribution on the PLLA fibers and good adhesion were believed to be achieved owing to electrostatic interaction forces between the fibers and ZnO NPs. Polymer fibers of PLLA produced by electrospinning are known to have a high negative surface charge [26], while the laser-generated ZnO NPs possess positive surface charge, as will be described below in Section 3.4.

### 3.3. Spectroscopic Data

#### 3.3.1. UV-Vis Spectra

**Figure 5:**
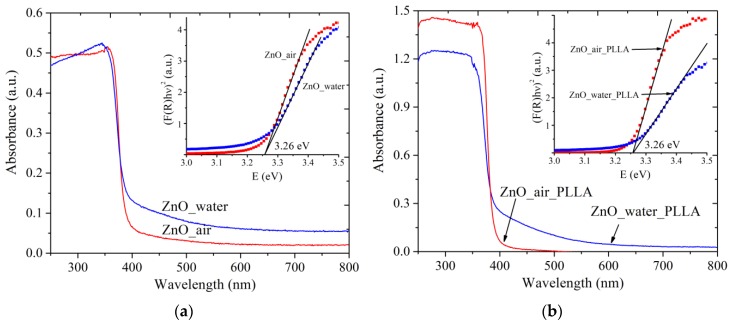
UV-Vis absorption spectra of powder samples (**a**) and ZnO-PLLA composites with NPs (**b**). Insets present how E_g_ values were evaluated. Red and blue lines represent data for ZnO NPs produced in air and water, respectively.

The absorption spectra of both powder samples are presented in Figure 5a. Similarly, Figure 5b exhibits absorption spectra of PLLA scaffolds loaded with the same NPs. The wurtzite ZnO phase is known to possess a characteristic shoulder of exciton absorption in the UV range below ~400 nm, which results from the electron transfer from the valence zone (formed by Zn(3d) and O(2p) orbitals) to the conductance zone (formed by Zn(4s) orbitals) [1,25]. This absorption band is observed at room temperature because of the high binding energy of excitons [1,17]. Both samples, ZnO_water (blue curve) and ZnO_air (red curve), are seen in panel (a) to have very weak absorption in the visible range, which is believed to result from admixtures (such as metallic Zn, carbonate species, and so on). It is because of such admixtures that sample ZnO_water was of grayish color. When the powders were loaded onto a polymer scaffold, the obtained composite materials are seen in panel (b) to demonstrate very similar spectra (compare blue and red curves in Figure 5a,b).

The absorption spectra were used to evaluate the bandgap of the materials (see insets in Figure 5). Interestingly, both samples gave the same value of ~3.26 eV, in both forms, i.e., as-prepared and after loading onto the PLLA matrix. This value agrees well with literature values for ZnO nanomaterials, where E_g_ was reported to be of 3.2–3.4 eV [1,27,28]. No quantum-confinement effects are observed as the obtained ZnO NPs are relatively large (over 10 nm).

#### 3.3.2. Photoluminescence Spectra

**Figure 6:**
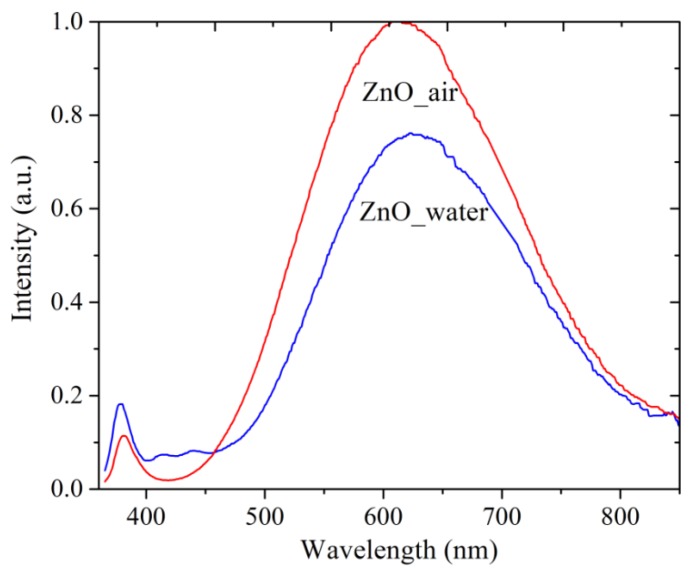
PL spectra of samples PLA-generated in air (red line) and water (blue). Excitation source use had λ_ex_ = 350 nm.

Figure 6 presents the PL spectra of as-prepared samples. Both samples (ZnO_air, red curve, and ZnO_water, blue curve) are seen to have two bands in their PL spectra: a narrow UV peak around 380 nm and a wide band in the visible range, with maxima at 612 nm (red spectrum) and 625 nm (blue spectrum). The two bands are known to have the exciton (UV) and defect (visible range) nature [6,15,16,29,30]. The maximum of defect-related visible PL of sample ZnO_water is less intense and red-shifted compared with that of sample ZnO_air. The more intense luminescence of sample ZnO_air is associated with a more defective structure of its NPs. The ZnO NPs prepared in air were shown to be smaller in size and, accordingly, they have a greater number of additional levels available for the radiative recombination of excited electrons. Another reason for a lower PL intensity demonstrated by sample ZnO_water is the presence of –OH groups on the surface of its NPs, as such groups are able to act as quenching centers of luminescence. 

It should be mentioned that there is no full agreement in the literature on interpreting defect-related PL in ZnO [15,16,29,30,31,32]. On one hand, the nature of the defects is obviously one of the main factors, while on the other hand, the NP size and morphology have also been reported to play a role [6,16,31,32]. Experimentally, it was found that during aging of ZnO colloids prepared via PLA in liquid, a red-shift of the defect-related PL band occurred [33]. According to [32], the redshift in ZnO PL is owing to the presence of defects such as Zn_i_, O_i_, and OH groups, and especially surface defects that are caused by all the peculiarities of PLA in liquid medium as preparation technique. Taking all of this into account, as well as the results of XRD and TEM presented above, one can conclude that the redshift of the PL band of sample ZnO_water could result from the following two factors: excess of O_i_ defects and the rod-shaped morphology of the NPs.

Altogether, the PL spectra in Figure 6 demonstrate that both powders produced by PLA are rich in defects, which should make them attractive for applications in catalysis or as antibacterial nanomaterials.

#### 3.3.3. IR and Raman Spectra

**Figure 7:**
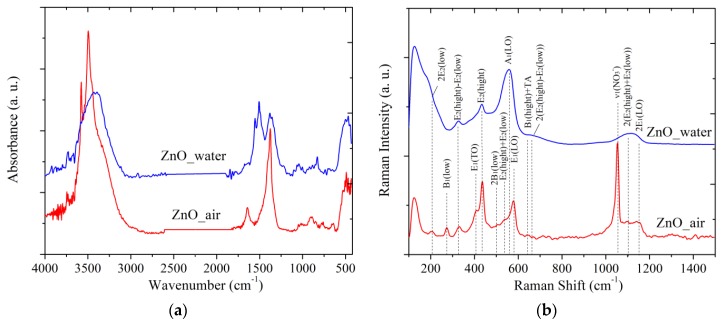
FTIR (**a**) and Raman (**b**) spectra of powder samples. Red and blue lines present data for samples prepared in air and water, respectively.

The IR spectra of the PLA-prepared samples are presented in Figure 7a. The band observed for both samples (red and blue curves) at 500 cm^−1^ is characteristic of valence vibrations of the Zn-O band [13]. Apart from Zn-O vibrations, two wide and intense bands in the range of 3750–2800 cm^−1^ and 1630–1200 cm^−1^ are observed for both samples, as well as several less pronounced bands between ~1000 and 600 cm^−1^.

Sample ZnO_water demonstrates a wide and featureless band at ~3400 cm^−1^, similar to what was previously reported by others for ZnO NPs prepared via PLA in water [9]. This band originates from the valent vibrations of OH groups [34], OH groups bound to H atoms [35], and from Zn-OH species [9]. In particular, there is a narrow peak at 3740 cm^−1^ which can be assigned to vibrations of isolated OH group of water on the [10 $\bar{1}0$] facet of ZnO [34]. The sample prepared in air exhibits more features in this spectral region (see red spectrum in Figure 7a). 

The second intense band of sample ZnO_water is believed to be related to carbonate species. For instance, the peak seen at 1372 cm^−1^ is most likely from the carbonate group of hydrozincite, which is also supported by another band at 1515 cm^−1^ [30]. The bands at 1049 and 825 cm^−1^ are also related to the υ_1_ and υ_2_ vibrations of carbonate groups from hydrozincite [36]. In this region, the sample PLA prepared in water demonstrates a more intense band at 1609 cm^−1^ which is related to deformational vibrations of intercalated H_2_O molecules. In addition, the vibration band υ_3_ of nitrate ions (1374 cm^−1^) belonging to Zn_5_(OH)_8_(NO_3_)_2_ × 2H_2_O is also expected to manifest itself in this region [37].

The Raman spectra of samples are exhibited in Figure 7b. The wurtzite ZnO (space group $C_{6v}^{4}$) is known to demonstrate the following optical modes: polar A_1_ and E_1_ that are active in the IR and Raman ranges, doubly degenerate E_2_ mode with frequencies E_2_(low) and E_2_(hight), and inactive “silent” B_1_ mode. The spectrum of sample ZnO_air (red line in Figure 7b) is seen to have more features, demonstrating, e.g., bands at 205, 408, and 437 cm^−1^, which are related to the main optical vibrations of the crystal lattice 2E_2_(low), transversal E_1_(TO) and E_2_(hight) vibrations, respectively. Since sample ZnO_air exhibits a more pronounced band E_2_(hight) at 437 cm^−1^, this implies that its NPs are better crystallized than those obtained in water (compare red and blue curves in Figure 7b). The band at 577 cm^−1^ can be assigned to either the A_1_(LO) or to E_1_(LO) lateral vibrations. This spectral range can also be linked to a mixed quasi-LO mode with E_1_ and A_1_ symmetries that are closely related to the presence of defects [38]. Because PLA-generated NPs are known to be rich in defects [7,9,15], it is safe to assume that the peak at 577 cm^−1^ originates from the E_1_(LO) mode. Finally, the bands below 150 cm^−1^ are hard to identify and are most likely to be related to defects as well. 

The distinctive feature of sample ZnO_water (blue spectrum in Figure 7b) is its wide dominating A_1_(LO) mode around 560 cm^−1^, which is characteristic of lattice vibrations parallel to the growth direction. This agrees well with the above presented TEM images where rod-like morphology of the NPs prepared in water was revealed. 

In agreement with the XRD results, where the presence of ZHN phase was revealed, the Raman spectrum of sample ZnO_air also demonstrates additional modes of this phase (see peaks at 273, 506 and 639 cm^−1^ in Figure 7b) that correspond to the B_1_(low), 2B_1_(low) and B_1_(hight)+TA vibrations, respectively. In addition, the intense peak at 1050 cm^−1^ belonging to υ_1_(NO_3_^−^) vibrations of nitrate ions is also observed [39]. The weak band at 713 cm^−1^ is also due to the nitrate ions, presening its υ_4_(NO_3_^−^) vibration mode. Finally, the wide band between 1050 and 1150 cm^−1^ observed for both samples is related to multi-phonon vibrations of ZnO crystal lattice.

Thus, the FTIR and Raman spectroscopic data correlate well with the above-presented XRD, PL and TEM results. In particular, they confirm: (i) the presence of ZHN phase in sample ZnO_air; (ii) the rod-shaped morphology of NPs generated in water; and (iii) the highly defective nature of both samples as previously revealed by their PL spectra.

### 3.4. Zeta Potential

**Figure 8:**
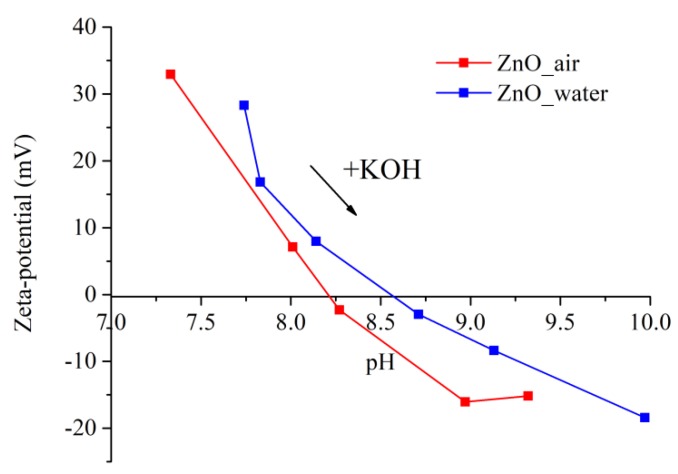
Zeta potential of NPs evaluated at different pH values. Blue and red symbols stand for samples prepared in water and air, respectively.

The value of ζ-potential is known to determine the stability of a colloidal solution, the system being stable when it is over ±30 mV. Information on the surface charge of NPs makes it possible to predict their interaction with bacterium cell membrane, the strength of which also depends on the bacteria type. Figure 8 exhibits the values of ζ-potential of the two samples dispersed in water as they were titrated with KOH solution to determine their pH_iep_.

It can be clearly seen in Figure 8 that the ζ-potential values of both samples are positive, being +33 mV (red curve) and +28 mV (blue curve) for NPs produced in air and water, respectively. Because of the larger value of its ζ-potential, the dispersion of sample ZnO_air is more stable in water, which is in agreement with the above discussion in Section 3.2.2. While the initial pH of water was ~6.3, the values were shifted to pH 7.3 (red curve) and pH 7.8 (blue curve) when the tested powders were dispersed. The values of the isoelectric point obtained for the dispersions were found to be in the basic range, agreeing well with the literature [40]. Thus, the surface of both types of NPs is positively charged at pH below this value, as protons from water are transferred onto the NPs and form surface Zn-OH_2_^+^ groups [41]. The difference between the two curves in Figure 8, and thus in the values of pH_iep_, implies that the surface chemistry of the two powders prepared in different media is different, which was previously revealed by both IR and Raman spectroscopy measurements.

As mentioned previously in Section 3.2.2, the positive ζ-potential of both laser-generated samples is an important factor that contributed to good surface adhesion and uniform deposition of their NPs onto PLLA scaffolds, the latter being negatively charged.

### 3.5. Antibacterial Activity

Table 2 shows the results of antibacterial tests obtained on the model PLLA tissues loaded with the two laser-generated ZnO powders. It can be clearly seen that both samples demonstrated good antibacterial activity towards *S. aureus* (A > 3, according to ISO 20743: 2013), with the activity of sample ZnO_air_PLLA being significantly higher. The latter finding could be explained by either the larger specific surface area and more defective structure of its NPs (with smaller sizes) or their surface chemistry; both assumptions require additional studies.

At the same time, the antibacterial activities of both samples towards *E. coli*, though exhibiting strong inhibitory effect, were found to be very close to each other, and lower than in the case of *S. aureus*. This agrees well with our previous results, reported for PLA-prepared ZnO NPs deposited onto cotton fabrics [10]. The observed difference in bactericidal action between the two strains tested is believed to be caused by their wall structures. While the *S. aureus* is known to be protected by its wall made of peptidoglycan (which has teichoic and lipoteichoic acids), the *E. coli* bacteria have an outer membrane that has a more complex chemical composition and contains lipopolysaccharide surrounded by a thin layer of peptidoglycan. The latter more complex structure provides the *E. coli* higher resistivity. It should be noted, however, that for wound dressing, it is the bactericidal action against the *S. aureus* that is of higher importance than that against *E. coli*.

There are several models explaining the antibacterial action of ZnO NPs. One possible mechanism proposed suggests that their bactericidal effect can be related to reactive oxygen species generated by such particles [42]. It cannot be excluded that the inhibiting action could also be due to the Zn^2+^ cations that are released as a result of partial dissolution of ZnO NPs [43] and result in membrane dysfunction [44] and NP internalization with the bacterium surface [45]. More details on possible bactericidal mechanisms of ZnO NPs can be found elsewhere [2,46]. 

The antibacterial tests carried out in this study confirm a high bactericidal potential of nano-sized ZnO loaded onto biodegradbalbe PLLA matrix as a model dressing for wound treatment. Besides, the novel ZnO powder produced via PLA in air was found to demonstrate higher antibacterial activity, which can be explained by its more uniform distribution over matrix fibers and by a larger specific surface area of its NPs.

## 4. Conclusions

In this study, we prepared ZnO nanoparticles (NPs) by ablating Zn target with nanosecond pulsed laser in water and air media and compared their properties. It was found that the NPs produced in air were of spherical shape, while their counterparts produced in water were somewhat larger and rod-shaped. Both samples were based on hexagonal wurtzite ZnO phase, while because of interaction with atmospheric nitrogen, the sample generated in air also had some fraction of monoclinic zinc hydroxynitrate. 

The NPs prepared in air were more stable in colloid (having zeta potential ζ > 30 mV) and demonstrated better dispersibility in water. After characterization by various structural and spectroscopic techniques, both powders were loaded onto polymeric matrix of biodegradable poly l-lactic acid, thus forming model biomedical composite materials for wound dressing. The antibacterial behavior of the two model dressings was tested against *S. aureus* and *E. coli* strains, showing promising bactericidal action against the former. 

Upon comparing ZnO NPs produced in water and air, this work demonstrates that the latter NPs had better dispersibility in water, while their antibacterial behavior was at least comparable with that of the former ones. Thus, when it comes to using ZnO NPs as powders, for example to disperse them and load them onto/into biodegradable polymer matrix, nanoparticles produced via laser ablation in air should be considered as real and promising alternatives to their counterparts prepared in water.

## Figures and Tables

**Table 1:** Structural characteristics and specific surface area values of prepared samples.

Sample	Phase Composition		Surface Area (m^2^/g)	NPs Average Size Parameters (nm)		
	Name	%		Diameter (nm)	Length (nm)	Width (nm)
ZnO_air	ZnO	92	36 ± 4	18–26	-	-
	ZHN	7				
	Zn	1				
ZnO_water	ZnO	>99	20 ± 2	12–21	30–100	14–20
	Zn	<1				

**Table 2:** Antibacterial activity of prepared samples towards *S. aureus* and *E. coli*.

Sample	The Level of Growth		Antibacterial Activity (A = F − G)
	Control F = lgC_t_ − lgC_0_	Sample G = lgT_t_ − lgT_0_	
*S. aureus* (+)			
ZnO_water_PLLA	+3.18	−1.62	+4.80
ZnO_air_PLLA		−2.48	+5.66
*E. coli* (−)			
ZnO_water_PLLA	+2.95	+1.53	+1.42
ZnO_air_PLLA		+1.67	+1.28
